# How to Tie a Surgeon’s Knot

**Published:** 1877-03

**Authors:** 


### HOW TO TIE A SURGEON'S KNOT.

A surgeon writes to the *Lancet* the following minute directions on this point:—

"Having placed your ligature under and across the artery, take a free end in each hand; keep the end in the right hand in front of the end in the left hand in crossing the string, so as to make it pass across, around and up under the end in the left hand; it will still be in front of the other free end, although it has changed hands; keep it in front in again bringing it across, and complete the knot in the usual way; the result would be a reef knot. If instead you pass the right hand end (which, after passing across, around, and up under the end in the left hand, is transferred as before to the left hand, and is still in front) behind the end that was in the left hand (but in making the knot has passed to the right hand), in completing the knot you will make a granny. Again, take an end in each hand, the end in the right hand being behind; keep it behind, and pass it, as before, across, around, and up under the end in the left hand; it will come up behind; still keep it behind, and on completing the knot a reef knot will be the result. If instead, before completing the knot, you allow it to pass in front of the other end, so altering its position with regard to its exit from around and under the other end, you will form a granny. In either case the knot is begun differently, and may be completed in two ways, a right and a wrong. The rule simply put, is: keep the end that comes up under (from around the other in tying the knot) always the same side. If it comes up in front of the other end, keep it in front of the other end in completing the knot. If it comes up behind, keep it behind, and you can not help making a reef knot; all that remains is to pull it tight.

